# Immunohistochemical evaluation of androgen receptor expression in endometrial carcinoma and its clinicopathological significance

**DOI:** 10.1186/s13000-026-01848-x

**Published:** 2026-09-26

**Authors:** Alaa saad Abd elhamid Mohamed, Heba Ashraf Hosni, Maram Salaheldeen Mahmoud, Alzahraa Abdelmoktader Mohammed

**Affiliations:** 1 Lecturer of pathology, Faculty of Medicine, Fayoum University, Fayoum, Egypt; 2 Lecturer of clinical oncology, Faculty of Medicine, Fayoum University, Fayoum, Egypt; 3 Lecturer of obstetrics and gynaecology, Faculty of Medicine, Fayoum university, Fayoum, Egypt

**Keywords:** Endometrial Carcinoma, Androgen Receptor, Immunohistochemistry, Biomarker, Clinicopathological Correlation

## Abstract

**Background:**

Endometrial carcinoma (EC) is a leading gynecological malignancy predominantly driven by hormonal imbalances. While the roles of estrogen and progesterone are well established, the influence of androgenic signaling, specifically through the androgen receptor, remains an underexplored diagnostic dimension. This study aims to evaluate the immunohistochemical (IHC) expression of the androgen receptor (AR) in EC tissue samples and correlate its presence with key clinicopathological parameters.

**Methods:**

A retrospective cohort study was conducted utilizing 40 formalin-fixed, paraffin-embedded (FFPE) tissue specimens from patients diagnosed with EC who had undergone total hysterectomies. Cases were histologically categorized according to World Health Organization (WHO) criteria into endometrioid endometrial carcinoma (EEC), *n* = 32 and serous endometrial carcinoma, *n* = 8, and classified by the International Federation of Gynecology and Obstetrics (FIGO) for staging and grading. IHC staining was performed to evaluate nuclear AR expression, defined as positive when > 10% of neoplastic cells exhibited distinct nuclear staining. Data were statistically analyzed to identify associations between AR status and patient age, histological subtype, tumor grade, and stage.

**Results:**

Distinct nuclear AR positivity was observed in 50% (20 of 40) of the total cohort. Expression was exclusively restricted to EEC (62.5% positive), whereas all serous carcinomas lacked expression entirely (*P* = 0.002). Notably, positive receptor expression demonstrated a strong inverse relationship with advanced FIGO stage and high tumor grade. Immunoreactivity was significantly enriched in early-stage disease (62.1% in Stages I and II versus 18.2% in Stage III; *P* = 0.013) and low-grade tumors (76.0% in Grades 1 and 2 versus 6.7% in Grade 3; *P* < 0.001). Patient age at diagnosis yielded no statistically significant correlation with expression status (*P* = 0.206).

**Conclusion:**

The AR is frequently expressed in low-grade, early-stage EEC, suggesting that AR may be associated with well-differentiated tumors and favorable clinicopathological parameters. The integration of AR immunohistochemistry into routine pathology panels could serve as a hypothesis-generating foundation for future clinical trials investigating targeted antiandrogen therapies.

## Introduction

Endometrial carcinoma (EC) represents the most frequently diagnosed gynecological malignancy in developed countries, with a steadily increasing incidence and mortality rate attributed to rising global obesity and metabolic dysfunction [1, 2]. Historically, EC has been classified into estrogen-dependent and hormone-independent categories based on distinct pathogenetic pathways [3]. However, contemporary diagnostic frameworks now integrate updated World Health Organization (WHO) classifications and comprehensive genomic characterizations established by The Cancer Genome Atlas to better predict tumor behavior, molecular subtypes, and guide precise clinical management [4, 5]. Specifically, this modern paradigm stratifies EC into four distinct molecular subgroups: POLE-ultramutated (POLEmut), mismatch repair-deficient (MMRd), p53-abnormal (p53abn), and no specific molecular profile (NSMP) [6]. Each subtype reflects a unique biological trajectory and therapeutic vulnerability [7]. The newly updated 2023 International Federation of Gynecology and Obstetrics (FIGO) staging system fundamentally restructured EC classification to formally mandate the integration of these genomic markers alongside traditional anatomical and histopathological findings [6].

While the proliferative effects of estrogen and the counter-regulatory mechanisms of progesterone are well-documented in EC pathogenesis, the influence of androgenic signaling remains a critical but underexplored dimension [8]. Large-scale randomization analyses and observational cohorts have recently demonstrated that elevated endogenous testosterone and bioavailable androgens significantly influence the risk profile for developing both endometrioid and non-endometrioid tumors [9, 10]. At the cellular level, these hormonal effects are mediated by the androgen receptor (AR), a nuclear transcription factor that regulates diverse physiological responses in female reproductive tissues [11].

Emerging functional evidence suggests that AR activation may actually exert a tumor-suppressive effect in the endometrium [12]. Specifically, recent in vitro and murine models have demonstrated that dihydrotestosterone (DHT) significantly suppresses EC proliferation, migration, and overall tumor growth directly through the AR pathway [12]. This protective mechanism is particularly relevant for postmenopausal women, where peripheral intracrine biosynthesis converts circulating precursors into bioactive androgens directly within the tumor microenvironment [13]. Furthermore, molecular profiling indicates that AR signaling can competitively antagonize the pro-proliferative transcription networks driven by estrogen, establishing AR as a crucial regulatory checkpoint in hormone-responsive tissues [14].

Despite these promising molecular findings, the true prevalence and distribution of AR expression in clinical EC tissue specimens remain unclear. Therefore, the objective of the present study is to evaluate the immunohistochemical (IHC) expression of AR in a cohort of EC tissue samples. By correlating AR expression status with key clinicopathological parameters, this study aims to clarify the diagnostic utility of AR and explore its potential role as a biomarker for tumor differentiation, advanced FIGO stage, and high tumor grade.

## Materials and methods

### Study design and patient cohort

This retrospective cohort study evaluated forty formalin-fixed, paraffin-embedded (FFPE) endometrial tissue specimens obtained from patients who underwent total hysterectomy at Fayoum University Hospital. Inclusion criteria were restricted to cases diagnosed as endometrioid or serous carcinomas that possessed sufficient well-preserved archival tissue for immunohistochemical staining. Other rare histotypes (e.g., clear cell, undifferentiated) were not encountered during the sampling period and were therefore excluded. The study protocol was reviewed and granted formal ethical approval by the Research Ethics Committee at the Faculty of Medicine, Fayoum University. Due to the retrospective nature of the study and the use of anonymized archival tissue, the requirement for direct informed patient consent was waived by the ethics committee. Comprehensive clinicopathological data, including patient age at diagnosis, histological subtype, tumor grade, and pathological stage, were extracted from the medical records.

### Histopathological evaluation

Standard histopathological processing was employed for all surgical specimens. Tissue blocks were sectioned at a thickness of 4 μm, mounted on electrostatically charged glass slides, and stained with routine Hematoxylin and Eosin (H&E). Histopathological diagnoses were established by two independent, board-certified pathologists who were blinded to the clinical data and outcomes. Tumors were classified according to the WHO diagnostic criteria into endometrioid endometrial carcinoma (EEC) and serous carcinoma subtypes. Disease staging and tumor grading were assigned in accordance with the International Federation of Gynecology and Obstetrics (FIGO) guidelines. For the purpose of statistical analysis, staging was grouped into low-stage (Stages I and II) and high-stage (Stage III) disease, while tumor differentiation was grouped into low-grade (Grades 1 and 2) and high-grade (Grade 3) categories.

### IHC staining protocol

IHC for the AR was performed on sequential FFPE tissue sections. Following deparaffinization in xylene and rehydration through a graded alcohol series, endogenous peroxidase activity was blocked by incubating the sections in a 3% hydrogen peroxide solution for 10 min. Heat-induced epitope retrieval (HIER) was subsequently conducted using a standard citrate buffer solution (pH 6.0) in a microwave oven for 20 min to unmask the antigenic sites.

After protein blocking to prevent non-specific binding, the tissue sections were incubated with a primary monoclonal mouse anti-human Androgen Receptor antibody (Clone AR441, Dako, Glostrup, Denmark) at a dilution of 1:50 for 60 min at room temperature. Immunodetection was carried out using a standard polymer-based secondary detection system. Chromogenic visualization was achieved using 3,3’-Diaminobenzidine (DAB), which precipitates as a distinct brown reaction product at the site of the target antigen. Finally, the slides were lightly counterstained with Mayer’s hematoxylin, dehydrated in ascending grades of alcohol, cleared in xylene, and permanently mounted. Tissue sections of benign prostatic hyperplasia (BPH) were included in every staining batch to serve as an external positive control. For negative controls, the primary antibody was omitted and replaced with phosphate-buffered saline (PBS).

### Evaluation of immunohistochemistry

The evaluation of AR immunostaining was conducted independently by two pathologists utilizing standard light microscopy. AR immunoreactivity was defined strictly by the presence of distinct brown nuclear staining within the neoplastic glandular epithelial cells. Cytoplasmic staining, if present, was disregarded. Cases were divided into AR-positive and AR-negative cohorts based on the presence of definitive nuclear staining, with tumors lacking nuclear expression entirely classified as negative. Cases were classified as AR-positive if > 10% of the neoplastic cell nuclei exhibited distinct brown staining.

### Statistical analysis

All statistical analyses were conducted utilizing IBM SPSS Statistics software, version 28.0 (IBM Corp., Armonk, NY, USA). Continuous clinical variables, specifically patient age, were assessed for normal distribution and expressed as the mean ± standard deviation (SD). Categorical variables, including histological subtype, FIGO stage, FIGO grade, and AR expression status, were presented as absolute frequencies and percentages. The associations between AR expression and clinicopathological parameters were evaluated using the Pearson Chi-square (χ2) test, or Fisher’s exact test where expected cell frequencies were insufficient. All statistical tests were two-sided, and a P-value of less than 0.05 was considered statistically significant.

## Results

### Patient demographics and clinicopathological characteristics

The current study evaluated formalin-fixed, paraffin-embedded (FFPE) endometrial tissue specimens obtained from 40 patients who underwent hysterectomies. Histological classification identified 32 cases (80%) as EEC and 8 cases (20%) as serous carcinoma. The mean age of the cohort at the time of diagnosis was 60 ± 9 years, with an age range spanning from 42 to 80 years.

Regarding disease staging and grading, the majority of the cohort presented with favorable clinicopathological parameters. According to the International Federation of Gynecology and Obstetrics (FIGO) criteria, 29 patients (72.5%) presented with low-stage disease (Stages I and II), whereas 11 patients (27.5%) were diagnosed with high-stage disease (Stage III). Similarly, histological grading revealed that 25 cases (62.5%) were low-grade (Grades 1 and 2), while 15 cases (37.5%) exhibited high-grade (Grade 3) features (Table 1).

### Associations between histological type and clinical parameters

Significant clinicopathological differences were observed between the two carcinoma subtypes. EEC predominantly affected younger patients, with 59.4% (*n* = 19) presenting at age 60 or younger, whereas 87.5% (*n* = 7) of serous carcinoma cases occurred in patients older than 60 years (*P* = 0.018). Furthermore, EEC was highly associated with early-stage disease; 87.5% of EEC patients presented with low FIGO stages compared to only 12.5% of those with serous carcinomas (*P* < 0.001) (Table 1).

**Table 1:** Clinicopathological characteristics of the study cohort and association with EC subtypes

Clinicopathological Variable	Total Cohort (*N* = 40) n (%)	EEC (*n* = 32) n (%)	Serous carcinoma (*n* = 8) n (%)	*P*-value
Age Category				0.018*
≤ 60 years	20 (50.0)	19 (59.4)	1 (12.5)	
> 60 years	20 (50.0)	13 (40.6)	7 (87.5)	
FIGO Stage				< 0.001*
Low (Stage I + II)	29 (72.5)	28 (87.5)	1 (12.5)	
High (Stage III)	11 (27.5)	4 (12.5)	7 (87.5)	
FIGO Grade				< 0.001*
Low (Grade 1 + 2)	25 (62.5)	25 (78.1)	0 (0.0)	
High (Grade 3)	15 (37.5)	7 (21.9)	8 (100.0)	

*EEC* Endometrioid Endometrial Carcinoma, *FIGO* International Federation of Gynecology and Obstetrics

* Indicates statistical significance (*P* < 0.05)

### AR expression and clinicopathological correlations

IHC analysis demonstrated distinct nuclear expression of the AR. Overall, AR positivity was observed in 20 out of 40 cases (50%). When classified according to histological subtype, AR expression was exclusively limited to EEC carcinomas. Specifically, 62.5% (*n* = 20) of EEC cases exhibited positive AR immunostaining, whereas all serous carcinoma cases (100%, *n* = 8) were completely devoid of AR expression (*P* = 0.002).

Robust associations were identified between AR positivity and favorable clinicopathological parameters (Table 2). Positive AR expression was significantly enriched in early-stage tumors, presenting in 62.1% (18/29) of low-stage cases compared to only 18.2% (2/11) of high-stage cases (*P* = 0.013). Similarly, AR immunoreactivity was strongly correlated with tumor differentiation; 76% (19/25) of low-grade tumors were AR-positive, contrasting sharply with only a 6.7% (1/15) positivity rate in high-grade tumors (*P* < 0.001). Patient age did not show a statistically significant correlation with AR expression status (*P* = 0.206).

**Table 2:** Correlation between AR Expression and Clinicopathological Variables

Clinicopathological Variable	Total (*N*)	AR Negative (*n* = 20) < br > n (%)	AR Positive (*n* = 20) < br > n (%)	*P*-value
Age Category				0.206
≤ 60 years	20	8 (40.0)	12 (60.0)	
≤> 60 years	20	12 (60.0)	8 (40.0)	
Histological Type				0.002*
EEC	32	12 (37.5)	20 (62.5)	
Serous carcinoma	8	8 (100.0)	0 (0.0)	
FIGO Stage				0.013*
Low (Stage I + II)	29	11 (37.9)	18 (62.1)	
High (Stage III)	11	9 (81.8)	2 (18.2)	
FIGO Grade				< 0.001*
Low (Grade 1 + 2)	25	6 (24.0)	19 (76.0)	
High (Grade 3)	15	14 (93.3)	1 (6.7)	

*AR* Androgen Receptor

*Indicates statistical significance (*P* < 0.05)

### IHC Findings

**Fig. 1:**
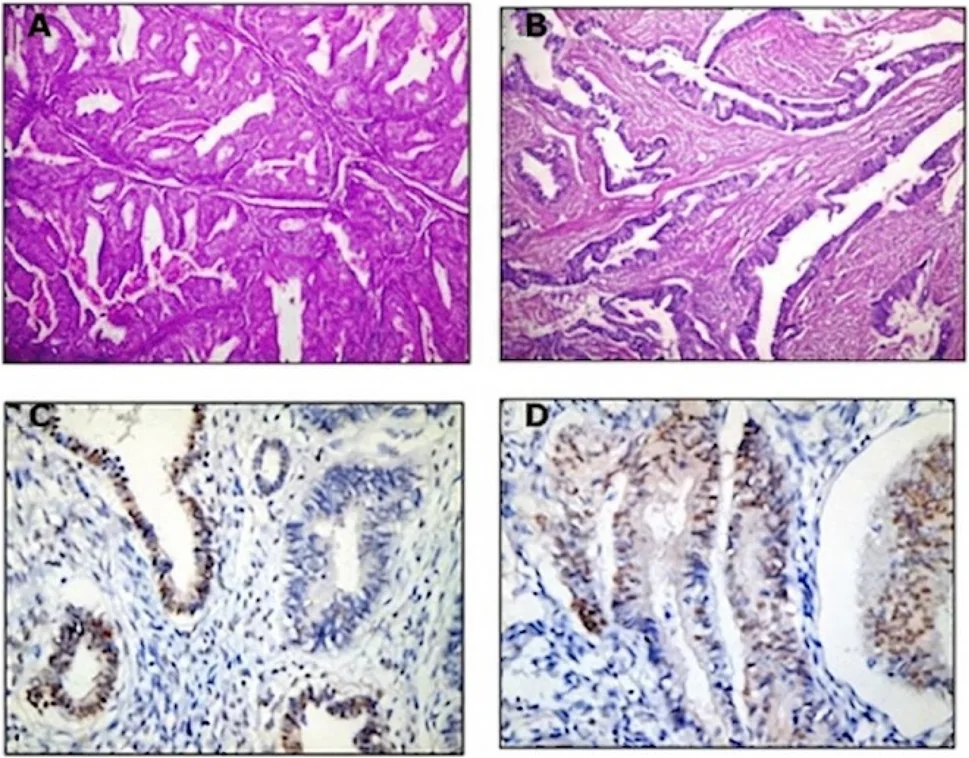
Histopathological and IHC features of low-grade EEC.** A**,** B** Standard H&E staining illustrating the classical architectural features of low-grade EEC at x100 and x200 magnification, respectively. **C**,** D** IHC staining of low-grade EEC utilizing AR antibodies, demonstrating robust and diffuse positive nuclear immunoreactivity within the neoplastic glandular epithelium (DAB, x200 and x400, respectively)

**Fig. 2:**
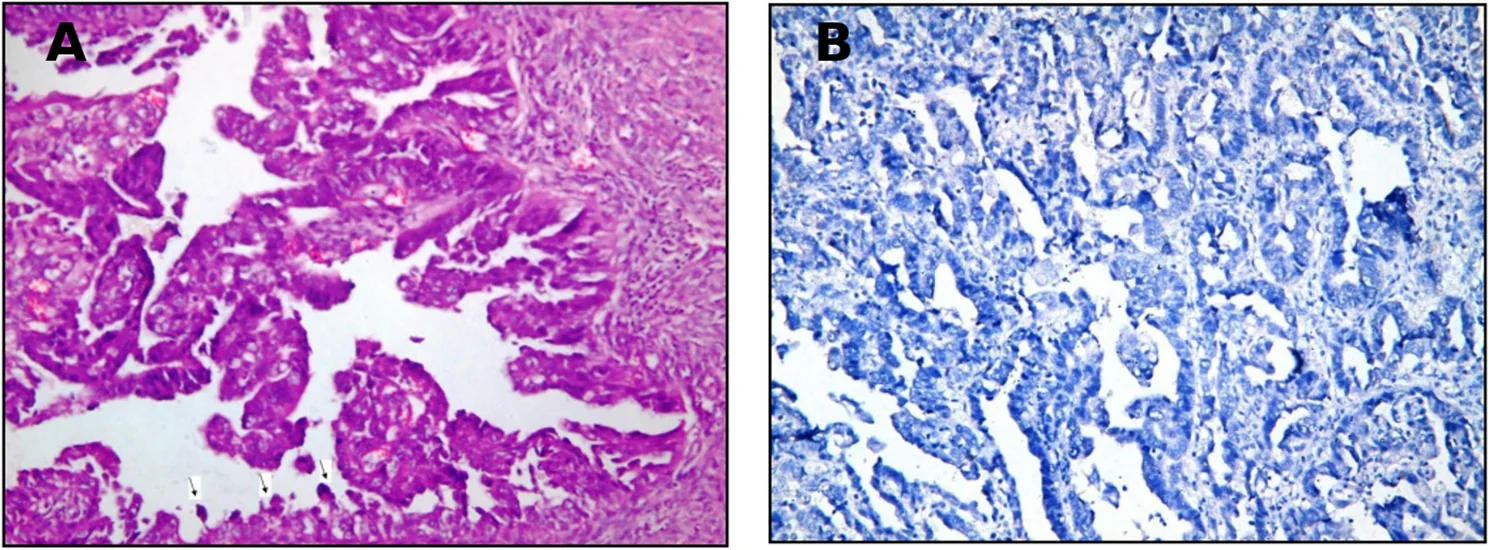
Histopathological and IHC evaluation of serous EC.** A** H&E staining of serous EC demonstrating characteristic high-grade cytological features, with arrows indicating prominent hobnailing (x200). **B** Corresponding IHC analysis of the same specimen utilizing AR antibodies, revealing completely negative immunostaining within the glandular epithelium (DAB, x200)

Microscopic evaluation confirmed strong, distinct nuclear staining for AR. Benign prostatic hyperplasia tissue utilized as a positive control demonstrated expected positive nuclear immunoreactivity. In low-grade EEC specimens, standard Hematoxylin and Eosin (H&E) staining revealed typical glandular architecture (Fig. 1A and B), while corresponding IHC staining confirmed robust positive nuclear AR expression within the neoplastic glandular epithelium (Fig. 1C and D). In contrast, histological examination of serous carcinoma revealed characteristic high-grade features, including prominent hobnailing (Fig. 2A), with complete absence of AR nuclear immunostaining in the glandular epithelium (Fig. 2B).

## Discussion

The present study evaluated the IHC expression of AR in EC and its association with clinicopathological variables. Our findings demonstrate an overall AR positivity rate of 50% (20/40 cases), with expression being exclusively restricted to EEC. Furthermore, AR positivity was significantly correlated with favorable clinicopathological parameters, including early FIGO stage and low histological grade, while no significant association was observed with patient age.

Our observed overall AR positivity rate of 50% aligns closely with several previous investigations. For instance, Mahdi et al. reported a 51% (135/261) positivity rate, and Zadeh et al. found AR expression in 54% (27/50) of their evaluated EC cases [15, 16]. Recently, Masoodi et al. reported a highly comparable AR positivity rate of 46% (23/50) in their analyzed cohort [17]. Providing definitive, large-scale validation of these figures, Viehweger et al. recently performed a massive IHC evaluation of over 18,000 tumors; among 200 EC specimens, 53% demonstrated positive AR staining [18]. However, a review of the broader literature reveals wide variability in AR detection rates. Hashmi et al. reported AR positivity in only 20.2% (18/89) of cases, whereas Moatamed et al. documented a markedly higher prevalence of 85% (60/71) [19, 20]. These discrepancies are likely attributable to methodological variations across institutions, including the use of different primary antibody clones, distinct scoring systems, and varying positivity thresholds (e.g., a 1% vs. 10% cutoff).

When stratifying by histological subtype, we observed a stark contrast: 62.5% of EEC were AR-positive, whereas 0% (0/8) of serous carcinomas in our cohort expressed AR (*P* = 0.002). This supports the conclusions of a comprehensive meta-analysis by Wu et al., which established that AR expression is significantly more prevalent in EEC (OR = 2.39, *P* < 0.001) [21]. Similarly, Tangen et al. reported a sequential decrease in AR positivity as tumors dedifferentiate, dropping from 74% in Grade 1 endometrioid tumors to 53% in Grade 3, and further down to 41% in non-endometrioid subtypes [22]. It is important to acknowledge, however, that conflicting data exists regarding serous carcinomas, whereas Zadeh et al. and Moatamed et al. reported high AR positivity (70% and 92%, respectively) in serous subtypes, and Hashmi et al. noted a 42.8% (3/7 cases) positivity rate in serous carcinomas [16, 19, 20]. Viehweger et al. also detected AR positivity in 61.1% of evaluated serous ovarian/ECs [18]. In addition, Cao et al. recently demonstrated robust AR expression across multiple aggressive histologies, detecting AR in 88% of serous carcinomas, 80% of carcinosarcomas, and 63% of undifferentiated/dedifferentiated ECs [23]. The complete absence of AR in our serous cohort heavily contradicts the high expression rates noted in larger studies. This discrepancy is almost certainly a limitation of our very small sample size for this subtype (*n* = 8), and it highlights the vast biological heterogeneity present within high-grade carcinomas. Therefore, our findings in the serous subgroup should be interpreted with extreme caution.

An important finding of our study is the strong inverse relationship between AR expression and advanced FIGO stage and high tumor grade. AR positivity was significantly enriched in low-stage tumors (62.1%) compared to high-stage cases (18.2%) (*P* = 0.013), as well as in low-grade (76%) versus high-grade (6.7%) tumors (*P* < 0.001). This corroborates the findings of Paudice et al., who demonstrated that average AR expression was significantly higher in low-grade endometrioid carcinomas (27.69%) compared to high-grade (11.0%) and high-risk histologies (15.6%) (*P* = 0.015) [24]. Likewise, Kamal et al. observed a significant reduction in epithelial AR expression in high-grade EC compared to low-grade EC (*P* < 0.0001) [25]. Similarly, Masoodi et al. documented that AR expression is prominent in hyperplastic and low-grade epithelium, but experiences a statistically significant loss as tumors progress to higher grades (*P* = 0.002) and more advanced stages [17]. In agreement, Cao et al. observed that diminished AR immunoreactivity was associated with worse clinicopathologic parameters in undifferentiated/dedifferentiated ECs [23]. A meta-analysis by Wu et al. further validated these associations, confirming that AR expression is strongly linked to low-grade disease (OR = 0.466, *P* < 0.001) and early FIGO stages I-II (OR = 0.480, *P* < 0.001) [21].

Regarding patient demographics, we observed no statistically significant correlation between AR expression and patient age (*P* = 0.206). This mirrors the findings of both Mahdi et al. and Paudice et al., who similarly reported that age at diagnosis does not influence AR status (*P* = 0.309 and *P* = 0.125, respectively) [15, 24].

The clinical relevance of AR in the endometrium is deeply rooted in its biological function. Evidence indicates that androgens exert a direct anti-proliferative effect on the normal endometrial epithelium [26]. In addition, normal endometrial physiology relies heavily on the intricate co-regulatory feedback between androgens, estrogens, and progesterone, wherein AR signaling actively antagonizes estrogen-driven cellular proliferation and facilitates apoptosis [27]. Consequently, the loss of AR expression may remove this critical inhibitory control, facilitating unrestrained, estrogen-driven tumor proliferation [12].

Our study is subject to several limitations. First, the relatively small sample size of 40 cases significantly limits the statistical robustness and generalizability of our findings. The subgroup analyses, particularly for the serous subtype, are underpowered, introducing a risk of Type II statistical error and limiting broad conclusions. However, previous large-scale studies have firmly linked AR loss to poorer survival metrics. Mahdi et al. demonstrated that patients with AR-positive tumors had a significantly longer mean survival of 208 months compared to 165 months for those with AR-negative tumors (*P* = 0.008) [15]. Correspondingly, Kamal et al. reported a significant reduction in disease-free survival among AR-negative patients (*P* = 0.0001), and Tangen et al. linked AR loss to significantly worse disease-specific survival (*P* < 0.001) [22, 25]. Recent prognostic evaluations further support this; Masoodi et al. provided contemporary evidence that AR positivity is significantly linked to prolonged disease-free survival (*P* = 0.024) [17].

Second, AR expression was evaluated as a binary variable based on a predefined threshold rather than a validated semi-quantitative scoring system (such as the H-score). This binary approach may obscure biologically relevant gradients of receptor expression.

Third, due to resource constraints, our study relied on traditional morphologic classification without the integration of modern TCGA molecular subgrouping (e.g., POLEmut, MMRd, p53abn). This omission limits the contemporary clinical relevance of our findings.

Additionally, critical clinicopathologic factors—including patient BMI, adjuvant treatment modalities, recurrence rates, and survival outcomes—were not available for analysis. Without long-term patient follow-up, we cannot make definitive claims regarding the true prognostic value of AR expression based on our cohort alone.

To address these limitations and build upon the current findings, future research should prioritize large-scale, prospective, multi-center studies to definitively validate the independent prognostic value of AR in EC. Furthermore, there is a critical need to establish universally standardized IHC scoring criteria and positivity thresholds for AR in gynecological pathology to ensure data reproducibility across different institutions.

Importantly, recent evidence highlights that a subset of histologically low-grade endometrioid tumors harbor p53 abnormalities, placing them in a high-risk category. Because p53 immunohistochemistry was not performed in our cohort, we cannot definitively exclude the overlap of AR-positive cases with p53-abnormal tumors. Future studies must evaluate AR alongside p53 to establish a more objective and clinically relevant framework. Furthermore, evaluating AR in isolation is insufficient to capture the complex hormonal interplay in endometrial carcinoma. Future investigations should utilize a multi-marker panel incorporating AR, Estrogen Receptor (ER), and Progesterone Receptor (PgR) to comprehensively profile the tumor’s biological behavior.

In conclusion, the AR is frequently expressed in early-stage, low-grade EECs, serving as a reliable biomarker for well-differentiated tumors associated with favorable clinicopathological parameters. The integration of AR immunohistochemistry into routine diagnostic panels could serve as a hypothesis-generating foundation for future clinical trials investigating targeted anti-androgen therapies.

## Data Availability

The datasets used and/or analysed during the current study are available from the corresponding author on reasonable request.

## Abbreviations

AR: Androgen receptor
BPH: Benign prostatic hyperplasia
DAB: 3,3’-Diaminobenzidine
DHT: Dihydrotestosterone
EC: Endometrial carcinoma
EEC: Endometrioid endometrial carcinoma
FFPE: Formalin-fixed, paraffin-embedded
FIGO: International Federation of Gynecology and Obstetrics
H&E: Hematoxylin and Eosin
HIER: Heat-induced epitope retrieval
IHC: Immunohistochemistry / Immunohistochemical
MMRd: Mismatch repair-deficient
NSMP: No specific molecular profile
OR: Odds ratio
p53abn: p53-abnormal
PBS: Phosphate-buffered saline
POLEmut: POLE-ultramutated
SD: Standard deviation
SPSS: Statistical Package for the Social Sciences
WHO: World Health Organization
